# Integrative profiling of Helicobacter pylori clinical isolates: virulence genes, antimicrobial susceptibility and genetic diversity in gastric cancer risk stratification

**DOI:** 10.1099/mgen.0.001599

**Published:** 2026-03-04

**Authors:** Ying Zhang, Yanyan Shang, Zhenkai Li, Zupeng Kuang, Shixuan Huang, Qinghua Ye, Jianhui Chen, Zhixin Huang, Ling Chen, Ying Li, Qingping Wu

**Affiliations:** 1 Department of Food Science and Engineering, School of Agriculture and Biology, Shanghai Jiao Tong University, Shanghai, 200240, PR China; 2 State Key Laboratory of Applied Microbiology Southern China, Guangdong Provincial Key Laboratory of Microbial Safety and Health, National Health Commission Science and Technology Innovation Platform for Nutrition and Safety of Microbial Food, State Administration for Market Regulation, Institute of Microbiology, Guangdong Academy of Sciences, Guangzhou, 510070, PR China; 3 Division of Gastrointestinal Surgery Center, the First Affiliated Hospital, Sun Yat-sen University, Guangzhou, 510080, PR China; 4 Food and Drug Laboratory, Guangdong Detection Center of Microbiology, Guangzhou, 510070, PR China

**Keywords:** biofilm formation, gastric cancer, genomic analysis, *Helicobacter pylori*, virulence genes

## Abstract

**Background*****.** Helicobacter pylori* infection is a major risk factor for gastric cancer (GC), especially in East Asia. However, the mechanisms through which bacterial strain heterogeneity contributes to GC risk remain poorly understood. This study aims to elucidate the roles of specific virulence factors, antibiotic resistance profiles and genetic variations in *H. pylori* in GC development.

**Methods.** We integrated host clinical data with phenotypic and genomic analyses of 31 clinical *H. pylori* isolates. Genomic analysis was performed to determine phylogenetic lineage and virulence markers. Antibiotic susceptibility and biofilm-forming ability of the clinical isolates were also assessed, and a genome-wide association study (GWAS) was employed to identify genetic polymorphisms linked to GC risk.

**Results.** Among the isolates, 93.5 % of isolates belonged to the East Asian lineage and carried high-risk virulence markers (*cagA* EPIYA-ABD, *vacA* s1m1). However, multidrug resistance was observed in 64.5 % of isolates, with high resistance rates to metronidazole (71.0%) and levofloxacin (48.4%) exceeding global averages. Strong biofilm-forming strains were significantly associated with tetracycline resistance compared to weak biofilm-forming strains. Additionally, GWAS identified five SNPs significantly associated with GC risk, with variants in *hemC*, *babB* and *C694_RS04850* being enriched in high-risk strains.

**Conclusions.** This study demonstrates the critical impact of *H. pylori* strain diversity in GC development, emphasizing the necessity for region-specific surveillance and innovative therapeutic strategies.

**Impact Statement**

This study reveals the high prevalence of East Asian lineage *Helicobacter pylori* strains carrying potent virulence markers and exhibiting multidrug resistance in a clinical cohort. It identifies significant associations between biofilm formation and antibiotic resistance and discovers novel genetic polymorphisms linked to gastric cancer risk. These findings provide critical insights for developing region-specific surveillance and personalized treatment strategies to mitigate *H. pylori*-associated gastric cancer.

## Data Summary

All supporting data have been provided within the article or through supplementary data files. Accession numbers of publicly available genome data are listed in Table S2 (PRJNA529500). The extracted data that support the findings of this study are available from the China Nucleotide Sequence Archive (CNSA, accession no.: CNP0008421).

## Introduction

*Helicobacter pylori* is a Gram-negative, microaerophilic bacterium that obligately colonizes the gastric mucosal surface, which has been classified as a group I carcinogen by the International Agency for Research on Cancer since 1994 [1,2]. Epidemiological evidence indicates that *H. pylori* is associated with 75 % of non-cardia gastric cancer (GC) and up to 98 % of cardia GC, both of which exhibit extremely poor prognoses, with 5-year survival rates below 20 % [3,5]. The high prevalence of *H. pylori* infection contributes significantly to GC morbidity and mortality [6]. Although global infection rates have declined to ~50 % due to sanitation improvements and eradication therapies [7,8], their prevalence remains geographically heterogeneous, disproportionately burdening low-resource regions [8,9]. Notably, only 10–20 % of infected individuals develop peptic ulcers, and merely 1 % progress to GC [3,10, 11], underscoring the critical role of strain-specific virulence determinants and host–microbe–environment interplay in disease outcomes.

The pathogenicity of *H. pylori* follows a multistep cascade: (1) urease-mediated pH buffering to modify the gastric microenvironment, (2) flagellar propulsion and helical morphology-driven chemotaxis through mucus layers, (3) adhesin-mediated epithelial attachment and (4) effector-triggered host cell damage via virulence factor secretion [12]. This process exhibits remarkable plasticity, shaped by dynamic interactions between bacterial genetic diversity and host susceptibility [13]. Critical virulence determinants, including cytotoxin-associated gene A (CagA), vacuolating cytotoxin A (VacA) and outer membrane proteins (OMPs), display marked inter-strain genetic variations that directly dictate pathogenic potential [3,10, 11, 14,17]. Such genetic diversity arises from high-frequency recombination and an elevated mutation rate (~2×10⁻⁵ per site/year during chronic infection), driving inter-strain divergence in gene content, virulence factor repertoire and sequence polymorphisms [18]. While allelic differences in core virulence genes significantly influence host interactions, the functional consequences of SNPs in pathogen–host interaction mechanisms remain poorly defined, which leaves a critical knowledge gap impeding risk stratification [18].

Another growing crisis in *H. pylori* management stems from escalating antibiotic resistance, particularly in the Asia-Pacific region [19]. Alarmingly high resistance rates of *H. pylori* are observed for clarithromycin (22% in Asia-Pacific; 52% in China) and levofloxacin (26% in Asia-Pacific; 47.2% in China), severely compromising treatment efficacy [19,21]. Furthermore, antibiotic overuse expands the intestinal microbiome resistome, accelerating horizontal acquisition of novel resistance genes that may modulate GC through yet-uncharacterized pathways [10,22, 23]. These trends necessitate urgent implementation of region-specific resistance surveillance systems and evidence-based antimicrobial stewardship programmes. Beyond genetic adaptation, *H. pylori* employs biofilm formation as an ecological survival strategy [24,25]. Biofilms enhance environmental persistence (e.g. water distribution systems [26] and food sources [27]) while conferring AMR tolerance and gastric colonization persistence [14]. Consequently, integrative analysis of virulence genotypes and biofilm phenotypes in clinical isolates is essential for deciphering disease progression patterns and prognostic stratification.

In this study, we established a multidimensional profiling of 31 *H. pylori* clinical isolates from Guangzhou, China. Critically, we established clinical correlations between these bacterial characteristics and gastric cancer risk. This study highlights the critical need to implement regional antimicrobial stewardship programmes and calls for active exploration of novel biocontrol approaches for *H. pylori* management.

## Methods

### Patients and samples collection

From July 2020 to July 2022, a total of 31 patients diagnosed with gastritis were enrolled at the First Affiliated Hospital of Sun Yat-sen University (Guangzhou, China). Gastric mucosal biopsy samples were collected from each participant during standard upper gastrointestinal endoscopy. The severity of gastritis and its potential risk of malignant transformation were evaluated independently by two experienced endoscopists using the Kyoto Gastritis Score (KGS) system [28]. Biopsy specimens were obtained from the gastric antrum (2–3 cm proximal to the pylorus). The collected biopsy specimens were immediately placed in sterile tubes, kept on ice and transported to the microbiology laboratory within 2 h [29].

This study was conducted in accordance with the Declaration of Helsinki and approved by the Ethics Committee of the First Affiliated Hospital of Sun Yat-sen University (approval no. 2020–164).

### *H. pylori* isolation and cultivation

The biopsy specimens were spread with sterile physiological saline and were cultured on the Columbia blood agar plates supplemented with 7 % defibrinated sheep blood (Huankai, Guangzhou, China) and commercial *H. pylori* selective supplement (Qingdao Rishui Biotechnology, Qingdao, China) [30]. Plates were incubated at 37 °C under microaerophilic conditions (5% O_₂_, 10 % CO_₂_ and 85% N_₂_) (Binder, Tuttlingen, Germany) for 3–7 days. Suspected *H. pylori* colonies were subcultured and confirmed by *16S rRNA* gene sequencing according to the method of Li [31]. Pure isolates were cryopreserved at −80 °C in Brucella broth containing 20% glycerol and 7% defibrinated sheep blood. All microbiological procedures were conducted in class II biological safety cabinets within BSL-2 containment facilities.

### Whole-genome sequencing and assembly

Genomic DNA from all 31 *H. pylori* isolates was extracted using a genomic DNA extraction kit (Huankai, Guangzhou, China) according to the manufacturer’s instructions. Genomic DNA underwent paired-end sequencing using the Illumina platform. In brief, libraries were prepared with the AMT Rapid DNA-Seq Kit (CISTRO, Guangzhou, China) and sequenced on a NextSeq 550 system (2×150 bp paired-end, Illumina, San Diego, CA, USA).

Raw reads were quality-filtered with Trimmomatic v0.39 [32] and *de novo* assembled with SPAdes v4.1 [33]. Assembly quality was comprehensively assessed with QUAST v5.2.0 [34] and CheckM v1.2.2 [35]. All genomes met standard bacterial-quality thresholds. The data that support the findings of this study have been deposited into China Nucleotide Sequence Archive (CNSA) [36] with accession number CNP0008421.

### Core-genome phylogenetics and population structure analysis

A globally representative dataset was constructed for phylogenetic and population structure analysis. We selected 310 genomes from the *Helicobacter pylori* Genome Project (HpGP; BioProject PRJNA529650) to capture the known genetic diversity across the 17 previously defined populations, thereby mitigating sampling bias (see Table S2 for geographic origins, available in the online Supplementary Material). These public genomes were combined with the 31 isolates newly sequenced in this study.

Core-genome SNPs (core SNPs) were identified by aligning all 341 genomes to the reference strain *H. pylori* ATCC 26695 (NC_000915) using Snippy v4.6.0 (https://github.com/tseemann/snippy). A maximum-likelihood phylogenetic tree was subsequently reconstructed from the core SNP alignment using FastTree v2.2 [37] under the general time-reversible model with a Gamma distribution to model site-specific rate heterogeneity.

For fine-scale population clustering, we employed the ChromoPainter (v0.04) [38]/fineSTRUCTURE (v0.02) [39] pipeline. The co-ancestry matrix generated by ChromoPainter, which infers DNA fragment transfers between haplotypes, served as the input for fineSTRUCTURE. The fineSTRUCTURE analysis was run for 100,000 Markov Chain Monte Carlo iterations following a burn-in period of 100,000 iterations to infer robust population clusters.

### Bioinformatic analysis of genetic determinants of virulence genes

The virulence genes of *H. pylori* in the Virulence Factor Database [40] were used as reference (Table S3). Except for the *dupA* gene from strain J99 (NC_000921), the reference strain for other virulence genes was strain 26695 (NCBI RefSeq: NC_000915). The presence of functional genes was defined with stringent thresholds of ≥90% nucleotide homology and ≥80% coverage. Heatmap visualization of virulence gene distribution was generated using R scripts (4.4.2) to reveal strain-specific pathogenicity patterns. The *vacA* and *cagA* gene sequences from the *H. pylori* isolates were extracted, aligned using MAFFT v7.525 [41], and subjected to allelic typing based on polymorphisms identified through multiple sequence alignment [42]. Variant identification was performed using the CLC Basic Variant Detection Module (QIAGEN) following the guidelines by Hadfield [43]. In brief, a reference seed sequence was used to perform local blastp (*e*-value ≤1e−5, identity ≥90%) against isolate sequences.

### Biofilm formation assay

Biofilm-forming capacity of the *H. pylori* isolates was quantified using crystal violet staining [27]. Briefly, *H. pylori* suspensions standardized to 1×10⁷ c.f.u. ml^−1^ were aliquoted and incubated at 37 °C under microaerobic conditions for 96 h. Planktonic cells were then removed, and adherent biofilms were washed, air-dried, stained with 0.1% (w/v) crystal violet and rinsed with PBS. The bound dye was dissolved in ethanol–acetic acid (95 : 5, v/v; 100 µl), and its absorbance was measured at 570 nm using a microplate reader (BioTek, Vermont, USA). Planktonic cell density (OD₆₀₀) was quantified from the aspirated suspensions to normalize biofilm biomass to bacterial growth. The biofilm unit (BU) was used to quantify the biofilm strength of the isolates, and the 31 isolates were classified into non-biofilm formers (BU <0.5), weak biofilm formers (BU: 0.5–1.0) and strong biofilm formers (BU >1.1).

### Antimicrobial susceptibility testing

The minimum inhibitory concentrations (MICs) of *H. pylori* against amoxicillin (AMX), clarithromycin (CLR), levofloxacin (LVX), metronidazole (MTZ) and tetracycline (TET) were determined via the agar dilution method, performed in accordance with the Clinical and Laboratory Standards Institute guidelines [44]. Briefly, bacterial suspensions were adjusted to 1×10⁷ c.f.u. ml^−1^ and inoculated onto the antibiotic-containing plates. MICs were determined after 72 h of incubation with quality control of *H. pylori* ATCC 43504. For clinical interpretation of resistance, breakpoints recommended by the European Committee on Antimicrobial Susceptibility Testing (v.15.0) were applied. MICs exceeding 0.125 mg l^−1^ for AMX, 0.25 mg l^−1^ for CLR, 1 mg l^−1^ for LVX, 8 mg l^−1^ for MTZ and 1 mg l^−1^ for TET indicated AMR of *H. pylori* [45].

### Genome-wide association study

To investigate the genetic variants associated with GC risk, a SNP-based genome-wide association study (GWAS) was performed using a linear mixed model implemented in GEMMA [46]. In brief, cleaned reads were aligned to the reference genome using BWA-MEM v0.7.17. Association testing was performed using a gemma-lmm 1 model, and genome-wide significance was determined using a threshold of *P*<1×10⁻⁵. Significant variants were functionally annotated using SnpEff v5.1 [47], and variants were classified based on sequence ontology terms, including synonymous, missense, nonsense and intergenic effects.

### Statistical analysis

The chi-squared test was applied to analyse differences in the biofilm production category and virulence factor profiles (*vacA* genotypes, *cagA* presence and antibiotic susceptibility). The Kruskal–Wallis one-way ANOVA with Dunn’s post hoc test was applied to analyse differences in urease activity between isolates, based on their biofilm-formation capacity. All tests were carried out using GraphPad Prism v8 (GraphPad, San Diego, USA) with a *P*-value of 0.05 or less as statistically significant.

## Results

### Demographic and clinical characteristics

This study enrolled a cohort comprised of 16 males (51.6%) and 15 females (48.4%), with ages ranging from 19 to 72 years. Gastroscopic evaluation assessed mucosal features including nodularity, diffuse redness, atrophy, intestinal metaplasia and enlarged folds (Table S1). Patients were stratified into low-risk (*n*=22) and high-risk (*n*=9) groups for GC based on the KGS (high-risk: score ≥4) [48]. Statistical analysis revealed no significant association between gender and GC risk (male, 9 low-risk vs. 7 high-risk; female, 13 low-risk vs. 2 high-risk; *P*=0.228). Although the ≥50-year subgroup exhibited a higher proportion of high-risk cases (5/12), age stratification (<40, 40–50, ≥50 years) showed no significant correlation with GC risk (*P*=0.46) (Table 1). These findings indicate that risk stratification in this cohort predominantly relied on endoscopic scoring rather than demographic factors.

**Table 1.** Kyoto gastrointestinal endoscopy classification and risk group distribution

Category	Subgroup	Total (*n*=31)	Low-risk group (*n*=22)	High-risk group (*n*=9)	*P*-value
**Gender**	Male	16	9(56.3%)	7 (43.7%)	0.077
	Female	15	13 (86.7%)	2 (13.3%)	
**Age**	<40 years	5	4 (80.0%)	1 (20.0%)	0.629
	≥40 and <50 years	14	11 (78.6%)	3 (21.4%)	
	≥50 years	12	7 (58.3%)	5 (41.7%)	

### Genetic diversity and phylogenetic analysis

**Fig. 1.**
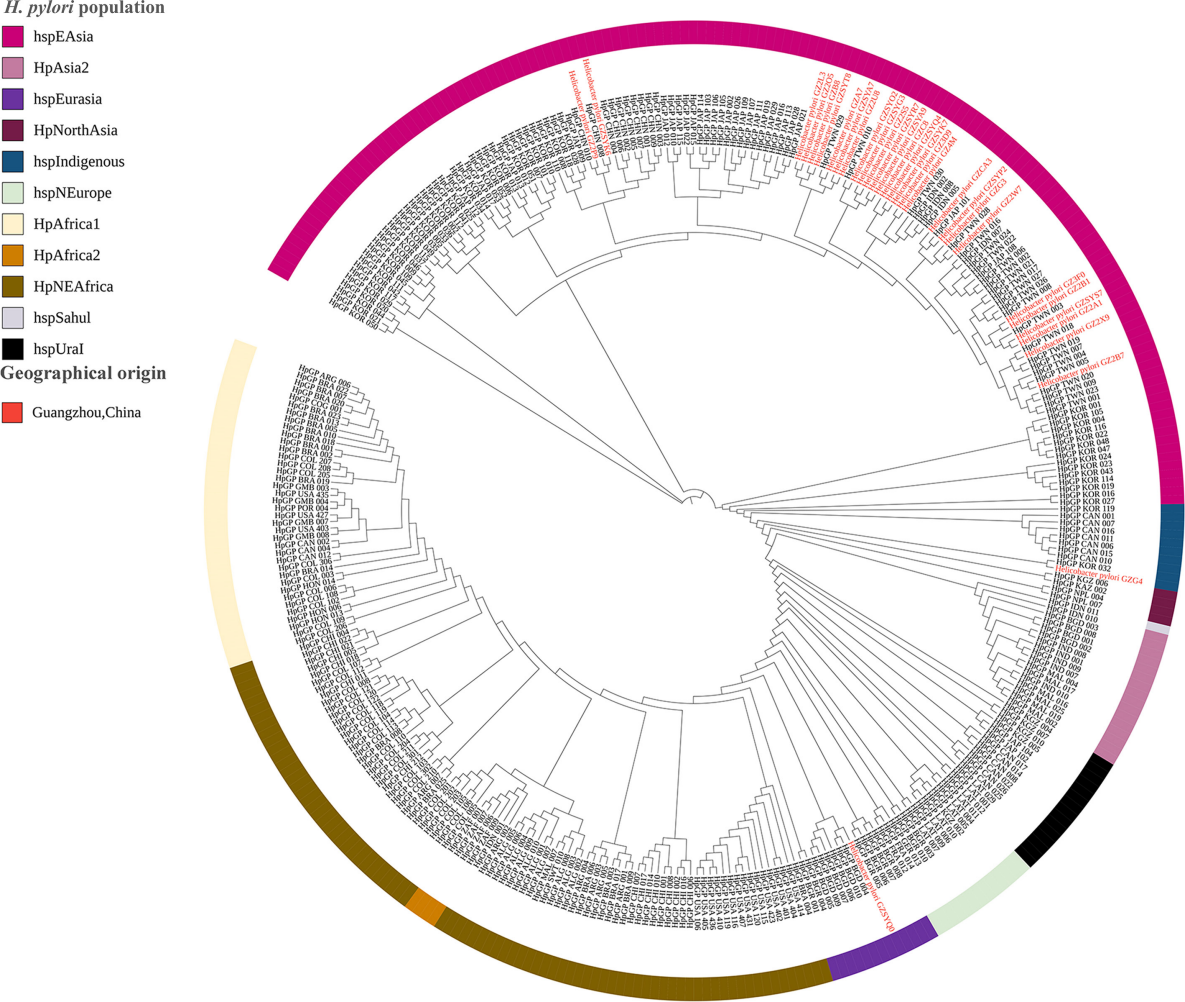
Phylogenomic architecture of Guangzhou strains. Phylogenomic architecture of the *H. pylori* strains was analysed based on core-genome SNP profiling. The maximum-likelihood phylogenetic tree was reconstructed using conserved SNPs across 31 clinical isolates from this study (marked in red) and 310 reference genomes from other studies (the colour of the outer ring indicates the *H. pylori* populations assigned in the phylogenetic analysis).

**Fig. 2.**
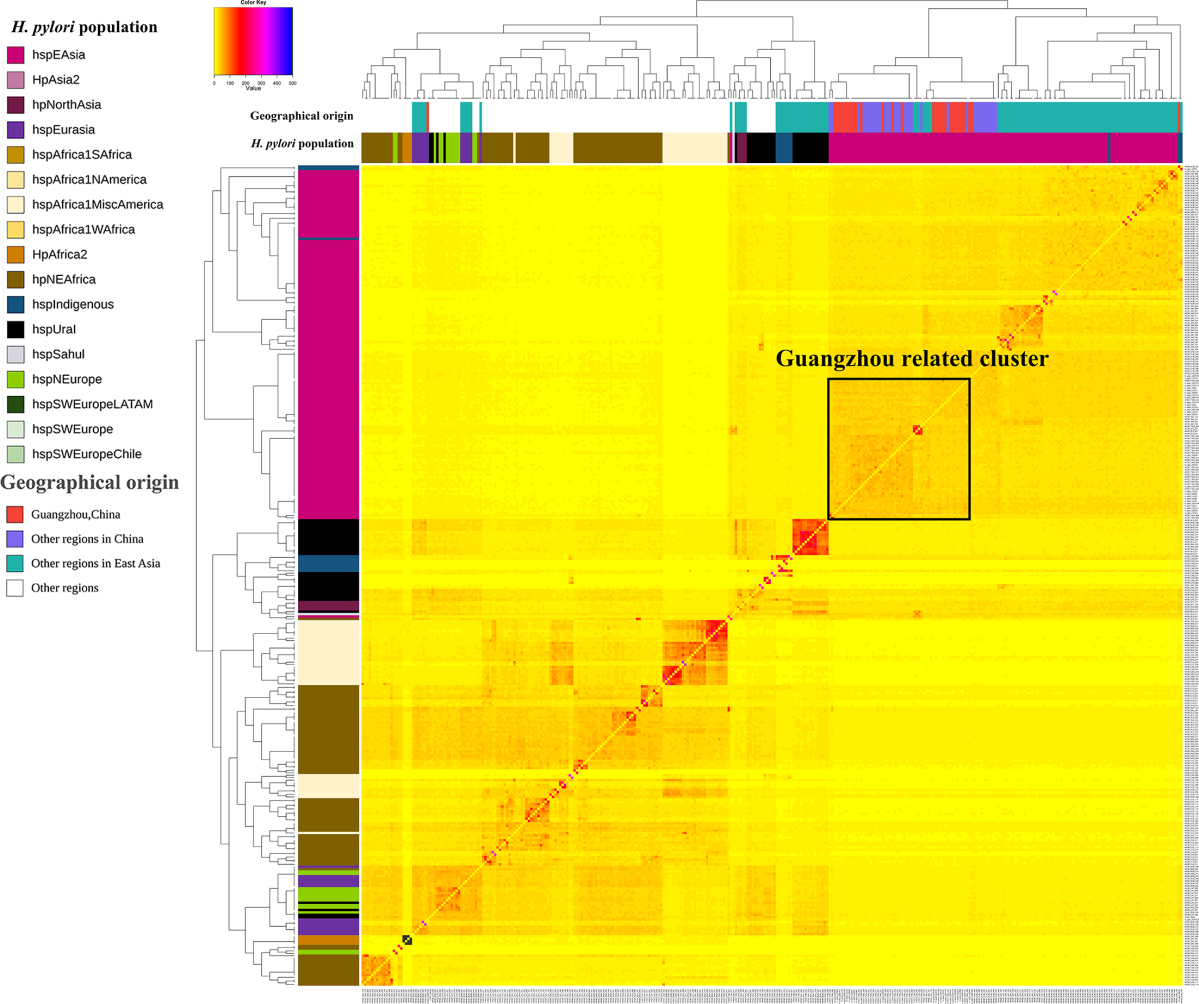
Population structure of *H. pylori* clinical isolates from Guangzhou, China. The population structure of the Guangzhou strains and the global published strains co-ancestry matrix was calculated using fineSTRUCTURE, including 31 Guangzhou strains in this study and 310 published strains. The columns on the top and left side show the different geographic regions and populations of the *H. pylori* strains with distinct colours. The colour of each cell of the matrix indicates the expected number of DNA chunks imported from a donor genome (column) to a recipient genome (row).

To elucidate the epidemiological characteristics of *H. pylori* in Guangzhou, we conducted a comprehensive phylogenetic analysis of 31 clinical isolates (Table S4) using core genome SNPs and co-ancestry matrix approaches (Figs 1 and 2). The phylogenetic framework incorporated 341 *H. pylori* genomes, comprising 310 globally published reference strains and 31 isolates from this study. Population structure analysis through clonal lineage relationships and fineSTRUCTURE classified these genomes into 11 and 17 primary populations and subpopulations, revealing distinct evolutionary stratification. The majority (93.5%, 29/31) of the clinical isolates in this study clustered within the hpEastAsia lineage and formed a unique Guangzhou-related cluster that was adjacent to other strains from China, showing strong phylogeographic specificity (Fig. 2). Notably, isolated from a 19-year-old male with chronic gastritis and duodenal ulcer (stage A2), *H. pylori* GZG4 was phylogenetically assigned to the rare hspIndigenous sublineage, which represents <1% of available East Asian genomes. Another outlier, *H. pylori* GZSYQ0, clustered within the hpEurasia lineage (includes the already reported hspCEurope/hspSEurope and hspMiddleEast) [49]. This isolate originated from a 32-year-old female in the low-risk group, presenting with chronic non-atrophic gastritis with erosion but no atrophy or intestinal metaplasia (Table S2). No isolates showed close phylogenetic relatedness to African lineages.

**Table 2.** Genetic variations associated with gastric cancer were identified by a GWAS comparing isolates from high-risk and low-risk patient groups

Position	REF	ALT	Variant type	GC risk (%)		Gene
				Low-risk	High-risk	
246842	T	C	Synonymous variant	5 (22.7%)	9 (100.0%)	*hemC*
948373	T	G	Synonymous variant	1 (4.5%)	6 (66.7%)	*babB*
1007182	G	A	Synonymous variant	2 (9.1%)	7 (77.8%)	*C694_RS04850*
1222671	C	T	Synonymous variant	21 (95.5%)	1 (11.1%)	*hopL*
1661028	A	G	Missense variant	21 (95.5%)	3 (33.3%)	*pdxJ*

### Virulence gene profiling

**Fig. 3.**
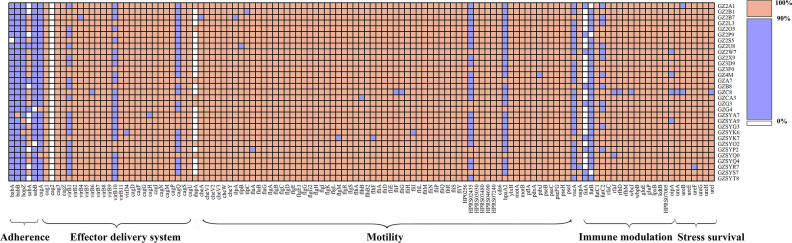
Virulence gene profile of *H. pylori* clinical isolates from Guangzhou, China. Heatmap visualization of virulence gene distribution across 31 clinical isolates. Colour key: orange, gene presence; white, gene absence; purple, low sequence similarity (<90 % identity). Virulence factors are hierarchically clustered by functional categories.

Virulence gene analysis of the Guangzhou clinical isolates revealed conservation of 76 core virulence genes, including acid resistance-associated urease gene clusters, motility-related flagellar biosynthesis genes and a functional type IV secretion system (T4SS) critical for toxin delivery (Fig. 3). Notably, five adhesion-related outer membrane protein genes (*hopS/babA*, *babB*, *hopZ*, *hopP/sabA* and *hopO/sabB*) exhibited significant sequence divergence (>80 % nucleotide variability). Further analysis focusing on toxin-encoding genes *vacA* revealed that all the 31 Guangzhou isolates harbored the highly virulent *vacA* s1m1 genotype. Similarly, analysis of the variable region in the C-terminal of CagA has identified the EPIYA-ABD motif in 93.5 % of strains, characteristic of East Asian *H. pylori* lineages. In contrast, non-Asian lineage strains GZG4 (hspIndigenous) and GZSYQ0 (hpEurasia) carried the characteristic motif of Western-type EPIYA-ABC motif, consistent with their phylogenetic divergence (Figs 1 and 2). Despite extensive virulence gene diversity, no direct associations were observed between specific virulence markers and GC risk stratification, likely reflecting functional redundancy among virulence factors or host-microenvironment modulation.

### Antibiotic resistance

**Fig. 4.**
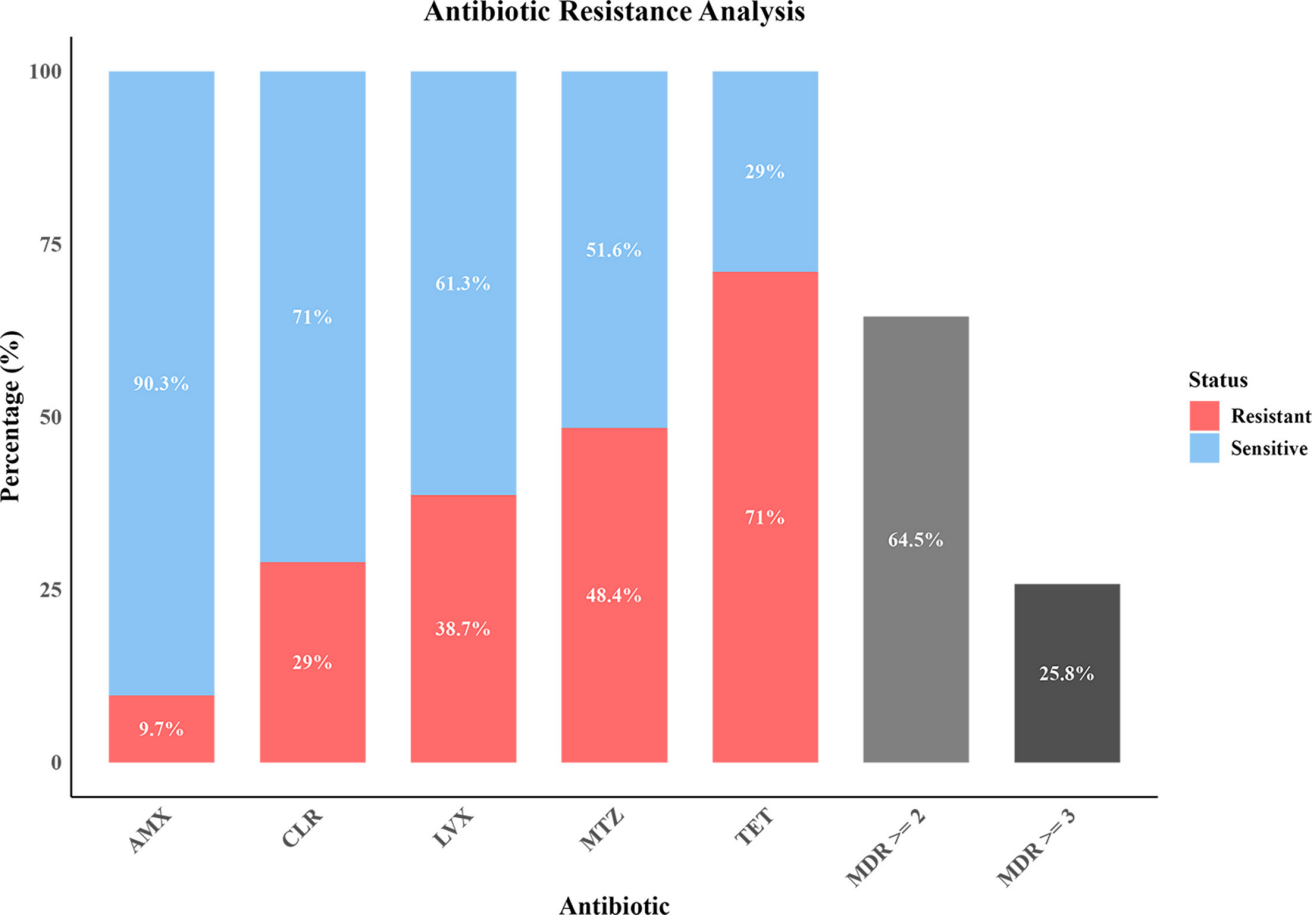
Bar-chart results of antibiotic resistance analysis. The chart shows the resistance rates of clinical isolates to five antibiotics (AMX, CLR, LVX, MTZ and TET) and the prevalence of multidrug resistance (MDR) to ≥2 or ≥3 antibiotics. The red and blue bars represent the proportion of resistant and susceptible strains, respectively.

The escalating antibiotic resistance of *H. pylori* poses a critical challenge to clinical management. Among the 31 clinical isolates, resistance rates were 38.7 % for CLR, 48.4 % for LVX, 29.0 % for AMX, 71.0 % for MTZ and 9.7 % for TET. Alarmingly, 93.55 % of the isolates demonstrated resistance to at least one antibiotic, with 64.5 % dual-drug resistant (resistant ≥2 antibiotics) and 25.8 % multidrug-resistant (resistant ≥3 antibiotics, Fig. 4). Notably, the Americas lineage strain GZG4 and European/Australian lineage strain GZSYQ0 exhibited quadruple resistance, underscoring the urgent need to establish region-specific antibiotic resistance profiles for tailored therapeutic strategies.

### Biofilm formation and its correlations with GC risk

**Fig. 5.**
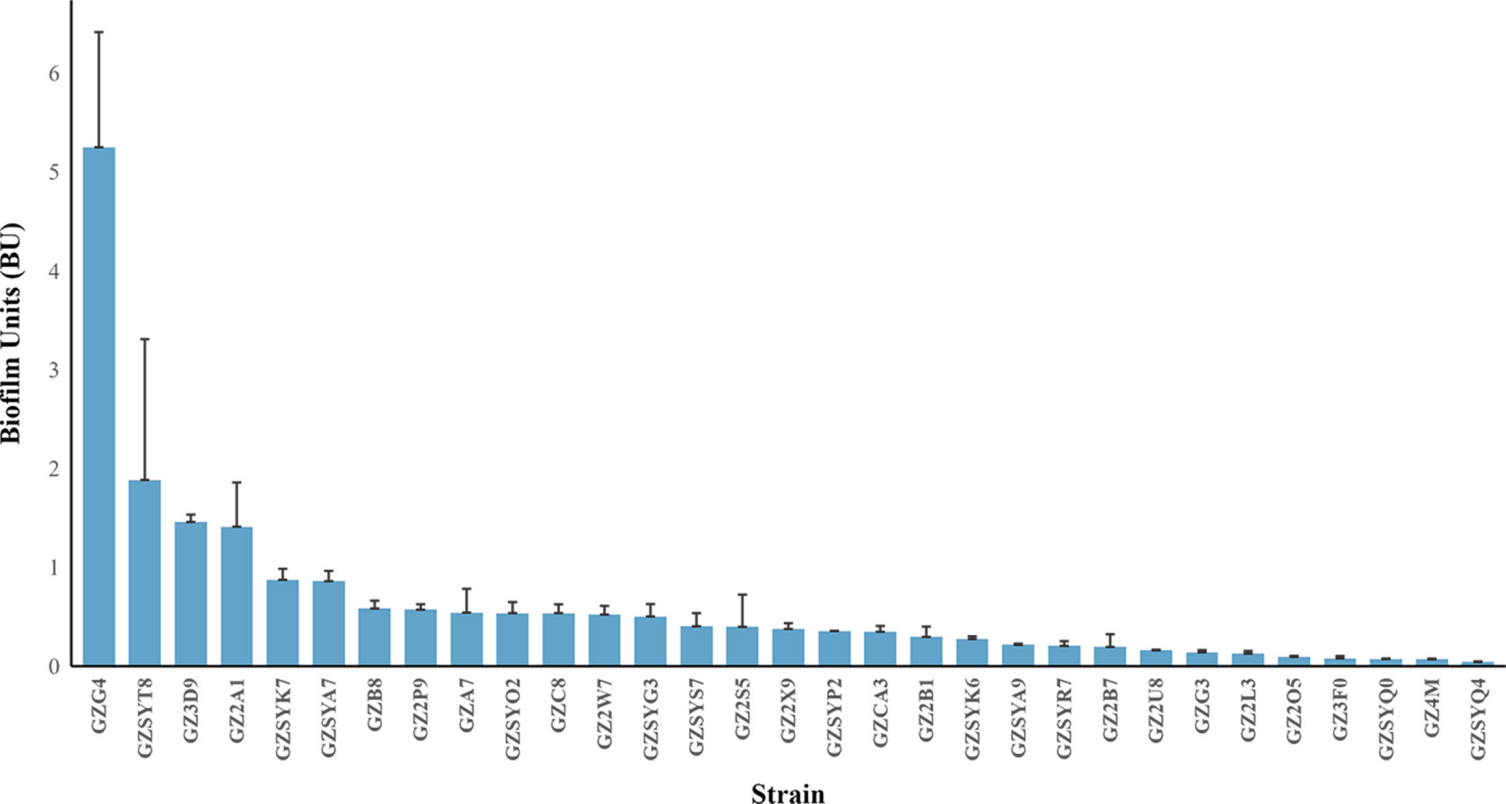
Biofilm formation of 31 Guangzhou strains. Biofilm quantification of clinical *H. pylori* isolates using the crystal violet assay.

Biofilm-forming capacity critically enhances *H. pylori* survival and antibiotic resistance [50]. In this study, biofilm phenotypes of 31 clinical isolates were comprehensively characterized using crystal violet staining (Fig. 5). Among these, 87.1 % (27/31) exhibited biofilm-forming ability, classified as strong (12.9%, 4/31) or weak (74.2%, 23/31) producers based on quantitative thresholds.

Notably, strong biofilm formers demonstrated significantly higher tetracycline resistance compared to weak/non-formers (75.4 % vs. 14.8 %, *P*=0.034), with a trend toward increased amoxicillin resistance (75.0 % vs. 22.2 %, *P*=0.067). No significant differences were observed for CLR, LVX or MTZ resistance between groups (*P*>0.05). Importantly, strong biofilm formation correlated significantly with endoscopic gastric atrophy (*P*=0.047; Table S3), suggesting biofilm-driven mucosal damage contributes to disease progression.

### GWAS

While previous studies have established that East Asia lineage backgrounds of *H. pylori*, such as *cagA*/*vacA* diversity and biofilm formation, collectively drive GC risk, these features fail to reliably stratify GC risk among the clinical isolates in this study [3,16, 17]. To address this limitation, we performed GWAS integrating host risk stratification, identifying five SNPs significantly associated with GC risk (Table 2). The Q–Q plot and Manhattan plot derived from the GWAS of 31 *H. pylori* isolates from Guangzhou are presented in Figs S1 and S2. These mutations were detected in *hemC* (porphyrin biosynthesis), *babB* (adhesion protein), C694_RS04850 (Na^+^/H^+^ antiporter) and *hopL* (outer membrane protein), while a missense mutation (A→G, position 1661028) was identified in *pdxJ* (pyridoxal 5′-phosphate biosynthesis). Notably, *babB* and *hopL* – both members of the OMP paralogous gene family – exhibited divergent risk associations: *babB* variants were enriched in high-risk strains (66.7%), whereas *hopL* mutations predominated in low-risk isolates (95.5%). Conversely, the *pdxJ* missense mutation showed a protective effect, with significant enrichment in the low-risk group (95.5%). Functional studies suggest that *pdxJ* dysfunction may impair flagellar glycosylation and metabolic adaptation, attenuating bacterial pathogenicity [51].

## Discussion

GC, the third leading cause of cancer-related mortality worldwide, exhibits a strong etiological association with chronic *H. pylori* infection. This is particularly evident in East Asia, which bears a disproportionately high burden of GC. This geographic clustering is associated with a high prevalence of *H. pylori* infection in the region and the circulation of phylogenetically distinct strains with enhanced virulence, such as the hspEAsia population [52,54]. This study systematically characterized the GC-inducing potential of *H. pylori* clinical isolates in Guangzhou, China, by integrating multidimensional data including endoscopic findings, genetic polymorphisms, virulence gene profiles, antibiotic resistance patterns and biofilm formation capacity. The investigation has identified novel biomarker combinations for predicting *H. pylori* strains with high GC risk.

*H. pylori* exhibits remarkable genetic diversity and plasticity, enabling its classification into distinct phylogeographic populations with defined global distributions [52]. These lineage-specific genetic signatures serve as molecular footprints to trace human migration histories [55]. Notably, the multidrug-resistant Amerind lineage strain GZG4 (hspIndigenous), isolated from a high-risk patient, harboured a Western-type EPIYA-ABC motif and quadruple antibiotic resistance (AMX, CLR, LVX and MTZ). This divergence underscores that pathogenicity is governed not merely by phylogeographic origin but through dynamic interplay between strain heterogeneity and host–environment interactions. Epidemiological studies have established associations between *H. pylori* virulence factors and gastric cancer [11,56, 57]. Several key virulence factors have been identified in *H. pylori*, including CagA, VacA and OMPs, all of which are implicated in gastric cancer development [3,16]. Comprehensive virulence network analysis revealed that all clinical strains harbored highly active *vacA* s1m1 variants, whose vacuolating toxin exacerbates mucosal damage by disrupting epithelial integrity and suppressing host immune responses [17,58]. However, despite 93.5 % of 31 Guangzhou-derived isolates carrying EPIYA-ABD-type *cagA* and 100 % expressing hyperactive VacA s1m1 toxin, no direct correlation existed between these high-virulence markers and patient gastric cancer risk stratification.

Furthermore, our investigation revealed alarmingly high resistance rates in Guangzhou *H. pylori* isolates, with MTZ and LVX resistance significantly exceeding Asia-Pacific regional averages (52% and 26 %, respectively, 1990–2022) [20]. This epidemiological pattern may be attributed to the historical overuse of nitroimidazoles and fluoroquinolones in South China. The high prevalence of multidrug-resistant *H. pylori* strains (64.5 % dual-resistant, 25.8 % triple-resistant) could lead to failure of standard therapy and persistence of chronic inflammation, ultimately leading to gastric lesions [59,60]. These findings underscore the urgent need for establishing regional antimicrobial resistance surveillance networks and implementing rapid molecular diagnostics to guide precision therapy [61,63]. Biofilm formation is also a key strategy employed by *H. pylori* to resist antibiotics. Consistent with previous reports [64], this study demonstrated that strong biofilm-forming strains exhibit higher resistance to TET and AMX than weak biofilm producers. This is likely attributable to the physical barrier provided by the biofilm matrix, which effectively impedes antibiotic penetration [59,60, 65]. In addition, biofilms enhance virulence and accelerate disease progression through two synergistic pathways: (1) enhancing bacterial colonization and drug tolerance and (2) sustaining chronic inflammation via persistent release of virulence effectors (e.g. urease and VacA) [17,66].

Following comprehensive profiling of virulence determinants, AMR patterns and biofilm formation dynamics in clinical isolates, this study focused on identifying GC-associated SNPs within high-risk *H. pylori* strains. These genetic signatures may drive pathogen phenotypic diversification through modulation of virulence effector secretion and host–microbe interaction networks [18,60, 67]. GWAS identified four synonymous mutations and one missense variant exhibiting significant divergence between high- and low-risk GC groups (*P*<1×10^−6^). Notably, while all isolates in this study harbored the *babB* gene, 66.7 % (6/9) of high-risk strains carried an identical critical mutation (T→G, position 948373). The functional implications of this *babB* mutation merit consideration within established molecular frameworks. Previous studies have confirmed that BabA-Le^b^ represents the most evident bacterial adhesin–receptor interaction in *H. pylori* [68]. Despite *babB* lacking direct Lewis B antigen-binding capacity, its allelic variants may regulate *babA* expression through recombination events (*babB* shows structural homology to *babA*) [68,69]. BabA-mediated adhesion activates the T4SS, facilitating the injection of CagA toxin into host cells and subsequent upregulation of pro-inflammatory factors (e.g. *IL*-8) and oncogenic factors [18,70]. The clinical relevance of *babB* genomic variation is further supported by population-level evidence demonstrating a significant association between *babB*-negative strains and gastric cancer [71]. In summary, our findings suggest that specific genetic variations, particularly in *babB*, may contribute to * H. pylori*-associated gastric carcinogenesis. However, the limited and unbalanced sample size of this exploratory GWAS necessitates caution in interpretation. These associations should be regarded as preliminary and require validation in larger, independent cohorts. Future investigations should employ molecular biology approaches to functionally characterize these SNPs and assess their potential utility in risk stratification models. Integrating structural biology with host–pathogen interaction studies may ultimately reveal novel therapeutic targets, advancing beyond conventional antibiotic strategies toward precision interventions against *H. pylori*-mediated oncogenesis.

## Supplementary material

10.1099/mgen.0.001599 Uncited Supplementary Material 1.

## Abbreviations

AMX: amoxicillin
BU: biofilm unit
CagA: cytotoxin-associated gene A
CLR: clarithromycin
GC: gastric cancer
KGS: Kyoto Gastritis Score
LVX: levofloxacin
MICs: minimum inhibitory concentrations
MTZ: metronidazole
OMPs: outer membrane proteins
TET: tetracycline
T4SS: type IV secretion system
VacA: vacuolating cytotoxin A
