# Supplementary material for: Integrative profiling of Helicobacter pylori clinical isolates: virulence genes, antimicrobial susceptibility and genetic diversity in gastric cancer risk stratification
Source: Microb Genom. 2026 Mar 4;12(3):001599. doi: 10.1099/mgen.0.001599 (PMC12959884; doi:10.1099/mgen.0.001599)
Supplement: Uncited Supplementary Material 1. [file mgen-12-01599-s001.pdf]

Supplementary Material

Integrative profiling of *Helicobacter pylori* clinical isolates: Virulence genes, antimicrobial susceptibility and genetic diversity in gastric cancer risk stratification

Ying Zhang [1,2†], Yanyan Shang [2†], Zhenkai Li [2†], Zupeng Kuang [2], Shixuan Huang [2], Qinghua Ye [2], Jianhui Chen [3], Zhixin Huang [3], Ling Chen [4*], Ying Li [2*], Qingping Wu [1,2*]

[1] Department of Food Science and Engineering, School of Agriculture and Biology, Shanghai Jiao Tong University, Shanghai, 200240, PR China;

[2] State Key Laboratory of Applied Microbiology Southern China, Guangdong Provincial Key Laboratory of Microbial Safety and Health, National Health Commission Science and Technology Innovation Platform for Nutrition and Safety of Microbial Food, State Administration for Market Regulation, Institute of Microbiology, Guangdong Academy of Sciences, Guangzhou, 510070, PR China;

[3] Division of Gastrointestinal Surgery Center, the First Affiliated Hospital, Sun Yat-sen University, Guangzhou, 510080, PR China;

[4] Food and Drug Laboratory, Guangdong Detection Center of Microbiology, Guangzhou, 510070, PR China;

***Corresponding author:**

Qingping Wu (wuqp203@163.com) | Ying Li (liying@gdim.cn) | Ling Chen (chenling@gdim.cn)

[†] These authors contribute equally to the article and should be considered as co-first authors.

Supplementary Tables

Table S1. Demographics and clinical characteristics of patients with sequenced *H. pylori* isolates

**Strain ID**	**Nodularity**	**Diffuse Redness**	**Atrophy**	**Intestinal Metaplasia**	**Fold Enlargement**	**Total Score**
GZ2A1	0	2	0	0	1	3
GZ2B1	0	1	2	1	0	4
GZ2B7	0	1	0	0	0	1
GZ2L3	0	2	2	1	0	5
GZ2O5	0	1	0	0	1	2
GZ2P9	0	2	2	1	1	6
GZ2S5	1	2	0	0	1	4
GZ2U8	0	2	0	1	1	4
GZ2W7	0	2	0	0	1	3
GZ2X9	0	1	1	1	0	3
GZ3D9	1	2	1	1	1	6
GZ3F0	1	2	0	0	0	3
GZ4M	1	2	0	0	0	3
GZA7	0	1	0	0	0	1
GZB8	0	2	0	0	1	3
GZC8	0	2	0	0	1	3
GZCA3	0	2	2	1	1	6
GZG3	0	1	0	0	1	2
GZG4	0	2	2	1	1	6
GZSYA7	0	1	0	0	1	2
GZSYA9	1	1	0	0	0	2
GZSYG3	1	1	0	0	0	2
GZSYK6	0	2	0	0	1	3
GZSYK7	0	1	0	0	1	2
GZSYO2	0	2	0	0	1	3
GZSYP2	0	1	0	0	0	1
GZSYQ0	0	2	0	0	1	3
GZSYQ4	0	1	0	1	0	2
GZSYR7	0	1	0	0	0	1
GZSYS7	0	1	0	0	0	1
GZSYT8	0	2	1	1	0	4

Table S2. The 310 reference genomes from other studies employed for constructing phylogenetic trees and population structure analysis of *H. pylori*

HpGP ID	Country	Scaffolds	Bases	CDS	Genes	GenBank Accession ID
HpGP-26695-ATCC	UK	1	1667920	1535	1580	CP079087
HpGP-ALG-001	Algeria	1	1631355	1514	1559	CP079086
HpGP-ALG-002	Algeria	1	1636597	1519	1564	CP079085
HpGP-ALG-003	Algeria	1	1653596	1524	1569	CP079084
HpGP-ALG-004	Algeria	1	1636089	1510	1555	CP079083
HpGP-ALG-005	Algeria	1	1563520	1450	1495	CP079093
HpGP-ALG-006	Algeria	1	1617933	1508	1553	CP079082
HpGP-ALG-007	Algeria	1	1612412	1487	1532	CP079081
HpGP-ALG-008	Algeria	1	1641827	1512	1557	CP079080
HpGP-ALG-009	Algeria	1	1584247	1464	1509	CP079079
HpGP-ALG-010	Algeria	1	1629562	1499	1544	CP079078
HpGP-ARG-001	Argentina	1	1624657	1495	1540	CP079077
HpGP-ARG-002	Argentina	1	1639097	1516	1561	CP080089
HpGP-ARG-003	Argentina	1	1614729	1487	1532	CP079076
HpGP-ARG-004	Argentina	1	1564050	1445	1491	CP079075
HpGP-ARG-005	Argentina	1	1621840	1496	1541	CP079074
HpGP-ARG-006	Argentina	1	1616607	1488	1533	CP079073
HpGP-ARG-007	Argentina	1	1696855	1570	1615	CP079072
HpGP-BGD-001	Bangladesh	1	1612523	1481	1526	CP079068
HpGP-BGD-002	Bangladesh	1	1620609	1505	1550	CP079067
HpGP-BGD-003	Bangladesh	1	1606958	1482	1527	CP079092
HpGP-BGD-004	Bangladesh	1	1682188	1549	1595	CP079066
HpGP-BGD-005	Bangladesh	1	1737942	1595	1640	CP079091
HpGP-BGD-006	Bangladesh	1	1565314	1451	1496	CP079065
HpGP-BGD-007	Bangladesh	1	1664652	1548	1593	CP079064
HpGP-BGD-008	Bangladesh	1	1564065	1442	1487	CP079063
HpGP-BGD-009	Bangladesh	1	1649213	1516	1561	CP079062
HpGP-BGD-010	Bangladesh	1	1565774	1442	1487	CP079061
HpGP-BGR-001	Bulgaria	1	1651693	1516	1561	CP079060
HpGP-BGR-003	Bulgaria	1	1686987	1551	1596	CP079059
HpGP-BGR-004	Bulgaria	1	1635625	1509	1554	CP079058
HpGP-BGR-005	Bulgaria	1	1722628	1565	1610	CP079057
HpGP-BGR-006	Bulgaria	1	1643011	1523	1569	CP079056
HpGP-BGR-007	Bulgaria	1	1683336	1550	1595	CP079055
HpGP-BGR-008	Bulgaria	1	1629022	1518	1563	CP079054
HpGP-BGR-010	Bulgaria	1	1604243	1485	1530	CP079053
HpGP-BRA-001	Brazil	1	1650493	1514	1559	CP079052
HpGP-BRA-002	Brazil	1	1649978	1511	1556	CP079051

HpGP-BRA-003	Brazil	1	1620699	1490	1535	CP079050
HpGP-BRA-004	Brazil	1	1609347	1483	1528	CP079049
HpGP-BRA-005	Brazil	1	1659707	1512	1557	CP079048
HpGP-BRA-006	Brazil	1	1674090	1541	1586	CP079047
HpGP-BRA-007	Brazil	1	1641034	1510	1555	CP079046
HpGP-BRA-008	Brazil	1	1640553	1507	1552	CP079045
HpGP-BRA-009	Brazil	1	1570607	1444	1489	CP079044
HpGP-BRA-010	Brazil	1	1676807	1535	1580	CP079043
HpGP-BRA-013	Brazil	1	1686871	1550	1595	CP079041
HpGP-BRA-014	Brazil	1	1691857	1536	1581	CP079040
HpGP-BRA-017	Brazil	1	1611056	1498	1543	CP079038
HpGP-BRA-018	Brazil	1	1660087	1519	1564	CP079037
HpGP-BRA-019	Brazil	1	1663543	1522	1567	CP079036
HpGP-BRA-020	Brazil	1	1622043	1500	1545	CP079035
HpGP-BRA-022	Brazil	1	1633843	1507	1552	CP079034
HpGP-BRA-023	Brazil	1	1636227	1513	1558	CP079033
HpGP-BRA-025	Brazil	1	1658306	1527	1572	CP079032
HpGP-CAN-001	Canada	1	1625325	1513	1558	CP079031
HpGP-CAN-002	Canada	1	1681404	1541	1586	CP079090
HpGP-CAN-004	Canada	1	1648710	1507	1552	CP079030
HpGP-CAN-006	Canada	1	1526349	1417	1462	CP079029
HpGP-CAN-007	Canada	1	1550050	1436	1481	CP079028
HpGP-CAN-008	Canada	1	1676873	1553	1598	CP079027
HpGP-CAN-010	Canada	1	1500054	1396	1441	CP079026
HpGP-CAN-011	Canada	1	1523599	1414	1459	CP079025
HpGP-CAN-012	Canada	1	1712659	1572	1617	CP079024
HpGP-CAN-014	Canada	1	1678426	1568	1613	CP079023
HpGP-CAN-015	Canada	1	1498341	1391	1436	CP079022
HpGP-CAN-016	Canada	1	1534483	1420	1465	CP079021
HpGP-CAN-017	Canada	1	1685229	1542	1587	CP079020
HpGP-CAN-022	Canada	1	1646971	1524	1569	CP079016
HpGP-CAN-025	Canada	1	1642263	1526	1571	CP079014
HpGP-CAN-026	Canada	1	1634610	1526	1571	CP079013
HpGP-CHI-001	Chile	1	1641691	1533	1578	CP079598
HpGP-CHI-002	Chile	1	1619936	1502	1547	CP079597
HpGP-CHI-004	Chile	1	1648441	1512	1557	CP079596
HpGP-CHI-005	Chile	1	1663842	1522	1567	CP079595
HpGP-CHI-006	Chile	1	1639194	1502	1547	CP079594
HpGP-CHI-007	Chile	1	1605929	1483	1528	CP079593
HpGP-CHI-008	Chile	1	1609626	1486	1531	CP079592
HpGP-CHI-010	Chile	1	1639546	1516	1563	CP082183
HpGP-CHI-011	Chile	1	1710266	1594	1639	CP079591
HpGP-CHI-014	Chile	1	1663539	1534	1579	CP079590
HpGP-CHI-016	Chile	1	1592973	1485	1530	CP079589

HpGP-CHI-017	Chile	1	1646264	1532	1577	CP079588
HpGP-CHI-018	Chile	1	1672193	1532	1577	CP079587
HpGP-CHI-021	Chile	1	1703098	1543	1588	CP079586
HpGP-CHI-022	Chile	1	1666738	1530	1575	CP079012
HpGP-CHI-212	Chile	1	1714691	1566	1611	CP079004
HpGP-CHN-001	China	1	1597930	1502	1547	CP079003
HpGP-CHN-002	China	1	1583850	1465	1510	CP079002
HpGP-CHN-003	China	1	1622740	1521	1566	CP079001
HpGP-CHN-004	China	1	1584715	1482	1527	CP079000
HpGP-CHN-005	China	1	1637091	1535	1580	CP078999
HpGP-CHN-006	China	1	1574747	1460	1505	CP078998
HpGP-CHN-007	China	1	1587630	1476	1521	CP078997
HpGP-CHN-008	China	1	1640011	1525	1570	CP078996
HpGP-CHN-009	China	1	1577879	1477	1522	CP078995
HpGP-CHN-010	China	1	1650269	1538	1583	CP078994
HpGP-COG-001	DR Congo	1	1630386	1496	1541	CP078993
HpGP-COL-003	Colombia	1	1695443	1561	1606	CP078981
HpGP-COL-005	Colombia	1	1662179	1528	1573	CP078979
HpGP-COL-006	Colombia	1	1664661	1516	1561	CP078978
HpGP-COL-008	Colombia	1	1665362	1526	1571	CP078977
HpGP-COL-102	Colombia	1	1687924	1539	1584	CP078975
HpGP-COL-103	Colombia	1	1638876	1508	1553	CP078974
HpGP-COL-104	Colombia	1	1647692	1511	1556	CP078973
HpGP-COL-105	Colombia	1	1594792	1483	1528	CP078972
HpGP-COL-106	Colombia	1	1600153	1474	1519	CP078971
HpGP-COL-107	Colombia	1	1664051	1534	1579	CP078970
HpGP-COL-108	Colombia	1	1664507	1522	1567	CP078969
HpGP-COL-109	Colombia	1	1711935	1582	1627	CP078968
HpGP-COL-110	Colombia	1	1676877	1548	1593	CP078967
HpGP-COL-112	Colombia	1	1676845	1531	1576	CP078966
HpGP-COL-113	Colombia	1	1661030	1521	1566	CP078965
HpGP-COL-114	Colombia	1	1663589	1525	1570	CP078964
HpGP-COL-118	Colombia	1	1697405	1579	1624	CP078961
HpGP-COL-119	Colombia	1	1638083	1512	1557	CP078960
HpGP-COL-120	Colombia	1	1676968	1550	1595	CP078959
HpGP-COL-121	Colombia	1	1666603	1534	1579	CP078958
HpGP-COL-122	Colombia	1	1672930	1537	1582	CP078957
HpGP-COL-201	Colombia	1	1591061	1465	1510	CP078956
HpGP-COL-202	Colombia	1	1592936	1469	1514	CP078955
HpGP-COL-203	Colombia	1	1625215	1491	1536	CP078954
HpGP-COL-204	Colombia	1	1648930	1530	1575	CP078953
HpGP-COL-205	Colombia	1	1660637	1522	1567	CP078952
HpGP-COL-206	Colombia	1	1634751	1509	1554	CP078951
HpGP-COL-207	Colombia	1	1663597	1526	1571	CP078950

HpGP-COL-208	Colombia	1	1668664	1527	1572	CP078949
HpGP-COL-210	Colombia	1	1640247	1508	1553	CP078947
HpGP-COL-211	Colombia	1	1679305	1529	1574	CP078946
HpGP-COL-301	Colombia	1	1653492	1525	1570	CP078945
HpGP-COL-302	Colombia	1	1699172	1557	1602	CP078944
HpGP-COL-306	Colombia	1	1669212	1532	1577	CP078941
HpGP-FRA-012	France	1	1642672	1531	1576	CP079554
HpGP-FRA-014	France	1	1655118	1543	1588	CP079552
HpGP-GER-013	Germany	1	1613794	1498	1543	CP078905
HpGP-GMB-003	Gambia	1	1620274	1470	1515	CP078896
HpGP-GMB-004	Gambia	1	1637745	1493	1538	CP078895
HpGP-GMB-007	Gambia	1	1681498	1532	1577	CP078894
HpGP-GMB-008	Gambia	1	1686815	1537	1582	CP078893
HpGP-HON-006	Honduras	1	1661292	1516	1561	CP079530
HpGP-HON-013	Honduras	1	1652401	1511	1556	CP079523
HpGP-HON-014	Honduras	1	1696080	1556	1601	CP079522
HpGP-IDN-001	Indonesia	1	1690436	1557	1602	CP078880
HpGP-IDN-002	Indonesia	1	1543558	1444	1489	CP078879
HpGP-IDN-005	Indonesia	1	1609427	1499	1544	CP078876
HpGP-IDN-007	Indonesia	1	1572735	1462	1507	CP078874
HpGP-IDN-008	Indonesia	1	1566738	1459	1504	CP078873
HpGP-IDN-010	Indonesia	1	1632885	1509	1554	CP078871
HpGP-IDN-011	Indonesia	1	1555685	1443	1488	CP078870
HpGP-IND-001	India	1	1615201	1508	1553	CP078869
HpGP-IND-007	India	1	1634213	1511	1556	CP078863
HpGP-IND-008	India	1	1629937	1508	1553	CP078862
HpGP-IND-009	India	1	1626935	1495	1540	CP078861
HpGP-IND-010	India	1	1604992	1482	1527	CP078860
HpGP-JAP-002	Japan	1	1562309	1468	1513	CP079478
HpGP-JAP-009	Japan	1	1621311	1495	1540	CP079477
HpGP-JAP-010	Japan	1	1574397	1469	1514	CP079476
HpGP-JAP-012	Japan	1	1578055	1472	1517	CP079475
HpGP-JAP-013	Japan	1	1602993	1495	1540	CP079474
HpGP-JAP-015	Japan	1	1584499	1483	1528	CP079473
HpGP-JAP-016	Japan	1	1611534	1504	1549	CP079472
HpGP-JAP-017	Japan	1	1646976	1523	1568	CP079471
HpGP-JAP-019	Japan	1	1576864	1485	1530	CP079470
HpGP-JAP-021	Japan	1	1555891	1457	1502	CP079469
HpGP-JAP-022	Japan	1	1572869	1466	1511	CP079468
HpGP-JAP-026	Japan	1	1573045	1469	1514	CP079467
HpGP-JAP-028	Japan	1	1606474	1497	1542	CP079466
HpGP-JAP-029	Japan	1	1579445	1478	1523	CP079465
HpGP-JAP-101	Japan	1	1571531	1473	1518	CP078834
HpGP-JAP-102	Japan	1	1630918	1506	1551	CP078833

HpGP-JAP-103	Japan	1	1543209	1453	1498	CP078832
HpGP-JAP-104	Japan	1	1629601	1498	1543	CP078831
HpGP-JAP-105	Japan	1	1555420	1437	1482	CP078830
HpGP-JAP-106	Japan	1	1594270	1475	1520	CP078829
HpGP-JAP-107	Japan	1	1585669	1476	1521	CP078828
HpGP-JAP-108	Japan	1	1561411	1446	1491	CP078827
HpGP-JAP-109	Japan	1	1585338	1476	1521	CP078826
HpGP-JAP-111	Japan	1	1563824	1467	1512	CP078824
HpGP-JAP-112	Japan	1	1578838	1475	1520	CP078823
HpGP-JAP-113	Japan	1	1609003	1490	1535	CP078822
HpGP-JAP-114	Japan	1	1620727	1510	1555	CP078821
HpGP-JAP-115	Japan	1	1606574	1483	1528	CP078820
HpGP-KAZ-002	Kazakhstan	1	1647055	1545	1590	CP078808
HpGP-KGZ-002	Kyrgyzistan	1	1725670	1589	1634	CP078806
HpGP-KGZ-004	Kyrgyzistan	1	1676629	1546	1592	CP078804
HpGP-KGZ-005	Kyrgyzistan	1	1633592	1495	1540	CP078803
HpGP-KGZ-006	Kyrgyzistan	1	1650441	1532	1577	CP078802
HpGP-KGZ-007	Kyrgyzistan	1	1682539	1570	1615	CP078801
HpGP-KGZ-010	Kyrgyzistan	1	1697762	1585	1631	CP078798
HpGP-KOR-001	South Korea	1	1639173	1512	1557	CP079464
HpGP-KOR-002	South Korea	1	1597612	1488	1533	CP079463
HpGP-KOR-004	South Korea	1	1578635	1466	1511	CP079462
HpGP-KOR-005	South Korea	1	1635019	1517	1562	CP079461
HpGP-KOR-006	South Korea	1	1591078	1492	1537	CP079460
HpGP-KOR-007	South Korea	1	1573924	1467	1512	CP079459
HpGP-KOR-008	South Korea	1	1573168	1481	1526	CP079458
HpGP-KOR-009	South Korea	1	1595446	1488	1533	CP079457
HpGP-KOR-010	South Korea	1	1582866	1481	1526	CP079456
HpGP-KOR-012	South Korea	1	1625995	1514	1559	CP079455
HpGP-KOR-013	South Korea	1	1620816	1514	1559	CP078797
HpGP-KOR-014	South Korea	1	1558002	1452	1497	CP078796
HpGP-KOR-016	South Korea	1	1575407	1464	1509	CP078795
HpGP-KOR-019	South Korea	1	1622433	1502	1547	CP078794
HpGP-KOR-020	South Korea	1	1648740	1535	1580	CP078793
HpGP-KOR-021	South Korea	1	1573564	1454	1499	CP078792
HpGP-KOR-022	South Korea	1	1566880	1456	1501	CP078791
HpGP-KOR-023	South Korea	1	1589076	1476	1521	CP078790
HpGP-KOR-024	South Korea	1	1621161	1494	1539	CP078789
HpGP-KOR-025	South Korea	1	1607001	1499	1544	CP078788
HpGP-KOR-027	South Korea	1	1623106	1496	1541	CP078787
HpGP-KOR-028	South Korea	1	1571838	1459	1504	CP078786
HpGP-KOR-029	South Korea	1	1599356	1488	1533	CP078785
HpGP-KOR-032	South Korea	1	1641244	1525	1570	CP079454
HpGP-KOR-033	South Korea	1	1613357	1503	1548	CP079453

HpGP-KOR-034	South Korea	1	1602931	1492	1537	CP079452
HpGP-KOR-035	South Korea	1	1581821	1476	1521	CP079451
HpGP-KOR-036	South Korea	1	1620264	1505	1550	CP079450
HpGP-KOR-037	South Korea	1	1637274	1505	1550	CP079449
HpGP-KOR-041	South Korea	1	1605825	1505	1550	CP079448
HpGP-KOR-042	South Korea	1	1593735	1483	1528	CP079447
HpGP-KOR-043	South Korea	1	1617008	1503	1548	CP079446
HpGP-KOR-044	South Korea	1	1608074	1498	1543	CP079445
HpGP-KOR-045	South Korea	1	1609731	1510	1555	CP079444
HpGP-KOR-046	South Korea	1	1611772	1481	1526	CP079443
HpGP-KOR-047	South Korea	1	1567034	1451	1496	CP079442
HpGP-KOR-048	South Korea	1	1574538	1459	1504	CP079441
HpGP-KOR-049	South Korea	1	1615428	1500	1545	CP079440
HpGP-KOR-050	South Korea	1	1611527	1492	1537	CP079439
HpGP-KOR-051	South Korea	1	1619460	1498	1543	CP078784
HpGP-KOR-052	South Korea	1	1558379	1446	1491	CP078783
HpGP-KOR-053	South Korea	1	1561159	1449	1494	CP078782
HpGP-KOR-101	South Korea	1	1628820	1505	1550	CP079438
HpGP-KOR-105	South Korea	1	1593957	1491	1536	CP079437
HpGP-KOR-109	South Korea	1	1610271	1493	1538	CP079436
HpGP-KOR-110	South Korea	1	1623221	1505	1550	CP079435
HpGP-KOR-111	South Korea	1	1637383	1512	1557	CP078781
HpGP-KOR-112	South Korea	1	1612791	1480	1525	CP078780
HpGP-KOR-114	South Korea	1	1614990	1501	1546	CP078778
HpGP-KOR-115	South Korea	1	1572338	1465	1510	CP078777
HpGP-KOR-116	South Korea	1	1659610	1542	1587	CP078776
HpGP-KOR-117	South Korea	1	1594961	1499	1544	CP078504
HpGP-KOR-118	South Korea	1	1620101	1497	1542	CP078503
HpGP-KOR-119	South Korea	1	1588944	1487	1532	CP078502
HpGP-LAT-001	Latvia	1	1667401	1543	1588	CP094499
HpGP-LAT-004	Latvia	1	1673764	1548	1593	CP079431
HpGP-LAT-005	Latvia	1	1680090	1556	1601	CP079430
HpGP-LAT-006	Latvia	1	1655677	1552	1597	CP094497
HpGP-LAT-007	Latvia	1	1672884	1534	1579	CP079428
HpGP-LAT-009	Latvia	1	1650243	1504	1549	CP079426
HpGP-LAT-010	Latvia	1	1605087	1484	1529	CP079425
HpGP-LAT-011	Latvia	1	1656614	1510	1555	CP078501
HpGP-LAT-012	Latvia	1	1642396	1530	1575	CP079424
HpGP-LAT-029	Latvia	1	1660539	1514	1559	CP079408
HpGP-MAL-002	Malaysia	1	1682359	1544	1589	CP079388
HpGP-MAL-004	Malaysia	1	1649360	1516	1562	CP079386
HpGP-MAL-007	Malaysia	1	1699103	1554	1599	CP079383
HpGP-MAL-016	Malaysia	1	1671907	1548	1593	CP079378
HpGP-MAL-017	Malaysia	1	1590675	1473	1518	CP079377

HpGP-MAL-019	Malaysia	1	1615796	1482	1527	CP079375
HpGP-MAL-025	Malaysia	1	1632926	1502	1547	CP079370
HpGP-NPL-004	Nepal	1	1666477	1524	1569	CP078467
HpGP-NPL-007	Nepal	1	1645201	1521	1566	CP078464
HpGP-POR-004	Portugal	1	1650708	1552	1597	CP079336
HpGP-SWT-010	Switzerland	1	1650513	1513	1558	CP079266
HpGP-TWN-001	Chinese Taiwan	1	1615426	1499	1544	CP079259
HpGP-TWN-002	Chinese Taiwan	1	1549439	1440	1485	CP079258
HpGP-TWN-003	Chinese Taiwan	1	1585696	1481	1526	CP079257
HpGP-TWN-004	Chinese Taiwan	1	1579874	1469	1514	CP079256
HpGP-TWN-005	Chinese Taiwan	1	1596199	1481	1526	CP079255
HpGP-TWN-006	Chinese Taiwan	1	1601009	1479	1524	CP079254
HpGP-TWN-007	Chinese Taiwan	1	1628677	1516	1561	CP079253
HpGP-TWN-008	Chinese Taiwan	1	1585459	1474	1519	CP079252
HpGP-TWN-009	Chinese Taiwan	1	1577218	1462	1507	CP079251
HpGP-TWN-010	Chinese Taiwan	1	1605925	1489	1534	CP079250
HpGP-TWN-016	Chinese Taiwan	1	1610545	1495	1540	CP079249
HpGP-TWN-017	Chinese Taiwan	1	1607953	1483	1528	CP079248
HpGP-TWN-018	Chinese Taiwan	1	1631856	1516	1561	CP079247
HpGP-TWN-019	Chinese Taiwan	1	1583891	1467	1512	CP079246
HpGP-TWN-020	Chinese Taiwan	1	1634172	1531	1576	CP079245
HpGP-TWN-021	Chinese Taiwan	1	1570870	1452	1497	CP079244
HpGP-TWN-022	Chinese Taiwan	1	1558101	1444	1489	CP079243
HpGP-TWN-023	Chinese Taiwan	1	1560258	1464	1509	CP079242
HpGP-TWN-024	Chinese Taiwan	1	1651761	1521	1566	CP079241
HpGP-TWN-026	Chinese Taiwan	1	1625379	1504	1550	CP078252
HpGP-TWN-027	Chinese Taiwan	1	1691672	1552	1597	CP078251
HpGP-TWN-028	Chinese Taiwan	1	1592424	1479	1524	CP078250
HpGP-TWN-029	Chinese Taiwan	1	1592254	1473	1518	CP078249
HpGP-TWN-030	Chinese Taiwan	1	1577177	1463	1508	CP078248
HpGP-USA-106	USA	1	1653583	1534	1579	CP078235
HpGP-USA-115	USA	1	1649338	1530	1575	CP078234
HpGP-USA-116	USA	1	1613308	1503	1548	CP078233
HpGP-USA-119	USA	1	1624533	1517	1562	CP078232
HpGP-USA-120	USA	1	1607311	1498	1543	CP078231
HpGP-USA-401	USA	1	1604353	1486	1531	CP078214
HpGP-USA-402	USA	1	1627168	1508	1553	CP078213
HpGP-USA-403	USA	1	1652865	1510	1556	CP078212
HpGP-USA-404	USA	1	1604853	1486	1531	CP078211
HpGP-USA-405	USA	1	1648239	1530	1575	CP078210
HpGP-USA-407	USA	1	1619587	1505	1550	CP078208
HpGP-USA-410	USA	1	1607365	1500	1545	CP078206
HpGP-USA-414	USA	1	1605620	1480	1525	CP078202
HpGP-USA-423	USA	1	1708388	1593	1638	CP078193

HpGP-USA-427	USA	1	1664784	1543	1588	CP078190
HpGP-USA-431	USA	1	1633596	1526	1571	CP078188
HpGP-USA-435	USA	1	1608050	1476	1521	CP078184
HpGP-USA-436	USA	1	1659076	1543	1588	CP078183
HpGP-ZAF-001	South Africa	1	1714499	1566	1611	CP078172
HpGP-ZAF-006	South Africa	1	1641225	1503	1548	CP078169
HpGP-ZAF-007	South Africa	1	1655871	1507	1552	CP078168
HpGP-ZAF-009	South Africa	1	1681930	1544	1589	CP078166

Table S3. Virulence genes of *H. pylori* in virulence factors database

VFclass	Virulence factors	Related genes	*H. pylori* 26695 chromosome (NC_000915)	*H. pylori* J99 chromosome (NC_000921)
Acid resistance	Urease	*ureA*	HP0073	jhp0068
		ureB	HP0072	jhp0067
		ureE	HP0070	jhp0065
		ureF	HP0069	jhp0064
		ureG	HP0068	jhp0063
		ureH	HP0067	jhp0062
		ureI	HP0071	jhp0066
Adherence	AlpB (*hopB*)	*alpB/hopB*	HP0913	jhp0849
	Blood group antigen binding adhesins	*babA/hopS*	HP1243	jhp0833
		babB/hopT	HP0896	jhp1164
	H. pylori adhesin A	*hpaA*	HP0797	jhp0733
	HopZ	*hopZ*	HP0009	jhp0007
	HorB	*horB*	HP0127	jhp0117
	Sialic acid binding adhesins	*sabA/hopP*	HP0725	jhp0662
		sabB/hopO	HP0722	jhp0659
	adherence-associated lipoprotein AlpA (hopC)	*alpA/hopC*	HP0912	jhp0848
Immune evasion	*Lipopolysaccharide* Lewis antigens	*futA*	HP0379	jhp1002
		futB	HP0651	jhp0596
		futC	HP0093	jhp0086
Immune modulator	Neutrophil-activating protein (HP-NAP)	*napA*	HP0243	jhp0228
	Outer inflammatory protein	*oipA/hopH*	HP0638	jhp0581
Motility	Flagella	*flaA*	HP0601	jhp0548
		flaB	HP0115	jhp0107
		flaG	HP0751	jhp0688
		flgA	HP1477	jhp1370
		flgB	HP1559	jhp1467
		flgC	HP1558	jhp1466
		flgD	HP0907	jhp0843
		flgE_1	HP0870	jhp0804
		flgE_2	HP0908	jhp0844
		flgG_1	HP1092	jhp0333
		flgG_2	HP1585	jhp1492
		flgH	HP0325	jhp0308
		flgI	HP0246	jhp0231

		flgK	HP1119	jhp1047
		flgL	HP0295	jhp0280
		flhA	HP1041	jhp0383
		flhB_1	HP0770	jhp0707
		flhB_2	HP1575	jhp1483
		flhF	HP1035	jhp0389
		fliA	HP1032	jhp0392
		fliD	HP0752	jhp0689
		fliE	HP1557	jhp1465
		fliF	HP0351	jhp0325
		fliG	HP0352	jhp0326
		fliH	HP0353	jhp0327
		fliI	HP1420	jhp1315
		fliL	HP0809	jhp0745
		fliM	HP1031	jhp0393
		fliN	HP0584	jhp0531
		fliP	HP0685	jhp0625
		fliQ	HP1419	jhp1314
		fliR	HP0173	jhp0159
		fliS	HP0753	jhp0690
		fliY	HP1030	jhp0394
		motA	HP0815	jhp0751
		motB	HP0816	jhp0752
		pflA	HP1274	jhp1195
Others	DupA	*dupA*	-	jhp0917-8*
		cag1	HP0520	jhp0469
		cag2	HP0521*	jhp0470
		cag3	HP0522	jhp0471
		cag4	HP0523	jhp0472
		cag5	HP0524	jhp0473
		cagC	HP0546	jhp0494
		cagD	HP0545	jhp0493
		cagE	HP0544	jhp0492
		cagF	HP0543	jhp0491
Secretion system	Cag PAI type IV secretion system	*cagG*	HP0542	jhp0490
		cagH	HP0541	jhp0489
		cagI	HP0540	jhp0488
		cagL	HP0539	jhp0487
		cagM	HP0537	jhp0485
		cagN	HP0538	jhp0486
		cagP	HP0536	jhp0484
		cagQ	HP0535	jhp0483
		cagS	HP0534	jhp0482
		cagT	HP0532	jhp0481

		cagU	HP0531	jhp0480
		cagV	HP0530	jhp0479
		cagW	HP0529	jhp0478
		cagX	HP0528	jhp0477
		cagY	HP0527	jhp0476
		cagZ	HP0526	jhp0475
		virB11	HP0525	jhp0474
	T4SS effectors cytotoxin-associated gene A	*cagA*	HP0547	jhp0495
Toxin	Vacuolating cytotoxin	*vacA*	HP0887	jhp0819

Except for the *dupA* gene from strain J99 (NC_000921), the reference strain for other virulence genes was strain 26695 (NCBI RefSeq: NC_000915).

Table S4. Assembly and quality assessment of the genomes of *H. pylori* clinical strains isolated in Guangzhou

Sample name	Assembly method	Sequencing depth	Assembly total length	Contig count	Largest contig	N50
GZ2A1	SPAdes	91.58	1637945	51	288124	142557
GZ2B1	SPAdes	170.23	1656656	100	272455	93963
GZ2B7	SPAdes	136.14	1679322	191	327537	109625
GZ2L3	SPAdes	128.01	1594239	60	298051	113122
GZ2O5	SPAdes	148.73	1639995	58	289583	69720
GZ2P9	SPAdes	159.09	1601332	52	329909	105096
GZ2S5	SPAdes	162.69	1594065	59	316868	109019
GZ2U8	SPAdes	239.96	1560738	52	211873	177131
GZ2W7	SPAdes	184.38	1619515	64	333899	69406
GZ2X9	SPAdes	182.88	1613241	44	338364	132754
GZ3D9	SPAdes	267.39	1579009	63	313715	121909
GZ3F0	SPAdes	177.83	1614267	33	289500	112043
GZ4M	SPAdes	127.15	1624486	157	339647	79176
GZA7	SPAdes	141.5	1625546	86	361529	103062
GZB8	SPAdes	41.8	1606968	77	173580	74671
GZC8	SPAdes	290.24	1717244	295	190931	56894
GZCA3	SPAdes	167.99	1652538	147	291453	114631
GZG3	SPAdes	282.73	1612852	39	279876	151007
GZG4	SPAdes	273.57	1603652	90	164341	51183
GZSYA7	SPAdes	365.8	1594611	38	358543	82388
GZSYA9	SPAdes	76.78	1608754	71	283494	145699
GZSYG3	SPAdes	284.88	1635930	51	343592	106570
GZSYK6	SPAdes	44.1	1568966	113	100445	35801
GZSYK7	SPAdes	33.62	1566924	67	143025	66133
GZSYO2	SPAdes	293.67	1631212	44	653441	166116
GZSYP2	SPAdes	265.55	1595703	124	291650	115199
GZSYQ0	SPAdes	274.1	1665141	79	158556	84491
GZSYQ4	SPAdes	32.12	1573693	36	243430	166324
GZSYR7	SPAdes	35.48	1602027	89	118885	37582
GZSYS7	SPAdes	232.77	1632496	69	300357	113821
GZSYT8	SPAdes	268.62	1642048	50	283014	158557

Supplementary Figure

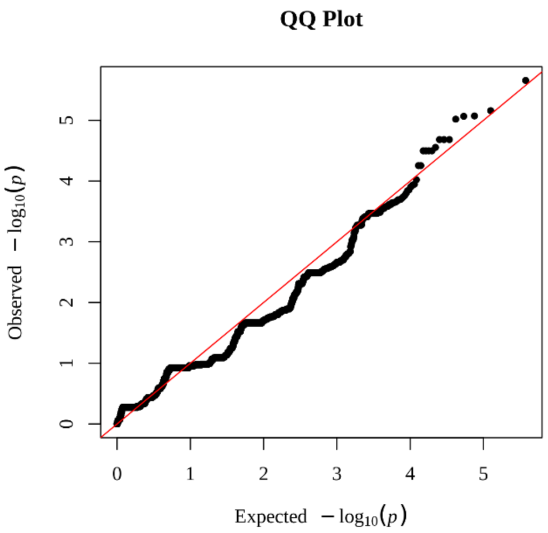

Figure S1. Q-Q plots of genome-wide association study (GWAS) tests between gastric cancer (GC) risk

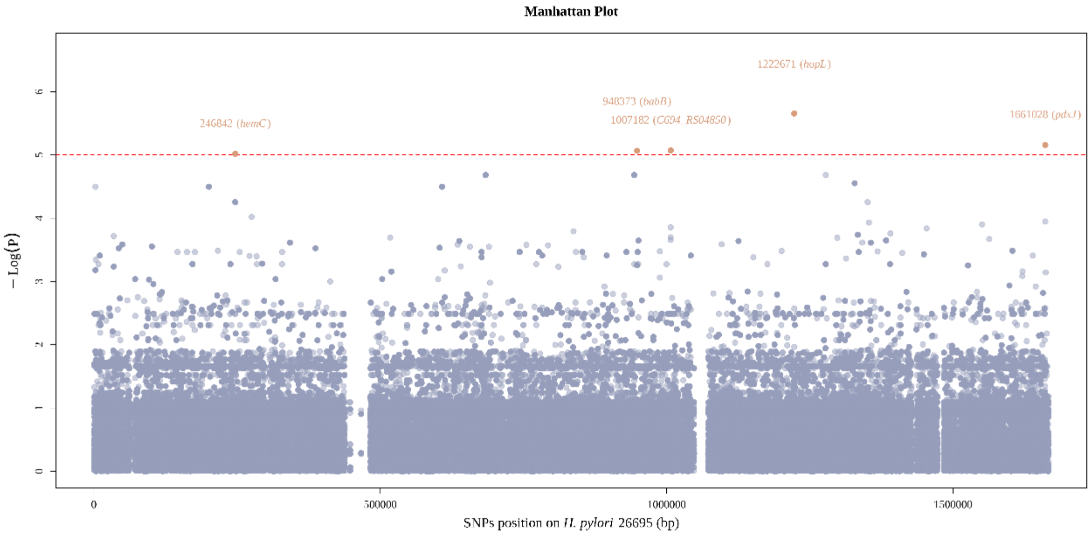

Figure S2. Manhattan plots of the genome-wide association study for gastric cancer (GC) risk of 31 *H. pylori* isolates from Guangzhou. Log_{10} (***P***) for each hit is recorded on the vertical axis. The gray dashed line indicates $P = 1x10^{-5}$.
